# Reduction of retinal vessel density in non-exudative macular neovascularization: a retrospective study

**DOI:** 10.3389/fmed.2023.1219423

**Published:** 2024-01-04

**Authors:** Yang Gao, Su Zhang, Yue Zhao, Tingting Yang, Paulo Moreira, Guangli Sun

**Affiliations:** ^1^Department of Ophthalmology, The First Affiliated Hospital of Shandong First Medical University & Shandong Provincial Qianfoshan Hospital, Jinan, Shandong Province, China; ^2^The Affiliated Eye Hospital, Nanjing Medical University, Nanjing, Jiangsu Province, China; ^3^International Healthcare Management Research and Development Center (IHM-RDC), Shandong Provincial Qianfoshan Hospital, Jinan, Shandong Province, China; ^4^Atlantica Instituto Universitario, Gestao em Saude, Oeiras, Portugal

**Keywords:** retinal image, non-exudative macular neovascularization, retinal blood flow, age-related macular degeneration, optical coherence tomography

## Abstract

**Purpose:**

The purpose of this study is to identify predictive activation biomarkers in retinal microvascular characteristics of non-exudative macular neovascularization (MNV) and avoid delayed treatment or overtreatment of subclinical MNV. The main objective is to contribute to the international debate on a new understanding of the role of retinal vessel features in the pathogenesis and progression of non-exudative MNV and age-related macular degeneration (AMD). A discussion on revising-related clinical protocols is presented.

**Methods:**

In this retrospective study, the authors included eyes with non-exudative MNV, eyes with exudative AMD, and normal eyes of age-matched healthy subjects. The parameters were obtained by optical coherence tomography (OCT) and optical coherence tomography angiography (OCTA).

**Results:**

In total, 21 eyes with exudative AMD, 21 eyes with non-exudative MNV, and 20 eyes of 20 age-matched healthy subjects without retinal pathology were included. Vessel density (VD) of the deep vascular complex (DVC) in eyes with non-exudative MNV was significantly greater than that in eyes with exudative AMD (*p* = 0.002), while for superficial vascular plexus (SVP) metrics, no VD differences among sectors were observed between eyes with non-exudative MNV and eyes with exudative AMD.

**Conclusion:**

The reduction in retinal vessel density, especially in the DVC, seems to be involved in or be accompanied by non-exudative MNV activation and should be closely monitored during follow-up visits in order to ensure prompt anti-angiogenic therapy. A discussion on applicable clinical protocols is presented aiming to contribute to new insights into ophthalmology service development which is directed to this specific type of patient and diagnosis.

## Introduction

Age-related macular degeneration (AMD) is the most common cause of visual impairment in industrialized countries (1–3). Macular neovascularization (MNV), the main characteristic of exudative AMD, leads to exudation, hemorrhage, and fibrosis formation and results in retinal damage and vision loss (1). João R. de Oliveira Dias et al. detected subclinical non-exudative MNV in 14.4% of non-exudative AMD eyes on OCT angiography (OCTA) among 160 patients with exudative AMD in one eye and non-exudative AMD in the fellow eye (4). It is known that the risk of developing exudation in eyes with non-exudative MNV was reported to be nearly 10 times higher than in eyes without these lesions (5). However, despite the higher risk of exudation, most experts recommended that prophylactic treatment before the exudation of non-exudative MNV should be avoided (6). Moreover, it has been suggested in protocols to preserve the potential protective and nutritional role of MNVs for the outer retina (7–9). Hence, to avoid delayed treatment or overtreatment, it is of great importance to find out the characteristics of non-exudative MNV that could serve as predictors of near-future exudation.

Some studies have tried to identify predictive activation biomarkers of subclinical non-exudative MNV, such as its size, shape, and growth rate (5, 10–12). However, to the best of our knowledge, there has not yet been any published study concerning retinal microvascular characteristics of non-exudative MNV on the risk of exudation.

In this study, we compared retinal vessel density and FAZ in non-exudative MNV and exudative AMD using OCTA to detect whether there is any difference in retinal blood flow between them and identify predictive activation biomarkers in retinal microvascular characteristics of non-exudative MNV, so as to ensure prompt anti-angiogenic therapy. This approach adds a new perspective on the role of retinal vessel density in the pathogenesis and progression of nonexudative MNV and AMD.

## Methods

In this retrospective study, we reviewed the charts of patients affected by treatment-naïve non-exudative MNV in AMD and exudative AMD who presented to the Affiliated Eye Hospital of Nanjing Medical University in China between April 2020 and December 2021.

The records were reviewed meticulously, including fluorescein angiography (FA), indocyanine green angiography (ICGA), and structural spectral-domain OCT (Heidelberg Engineering, Heidelberg, Germany). Two masked retinal specialists (YG and SZ) classified MNVs into three subtypes (1, 2, or 3) and determined the MNV activity status (exudative vs. non-exudative).

We excluded eyes with polypoidal choroidal vasculopathy (PCV), eyes with previous treatments, and eyes with any disease potentially affecting the image interpretation. Patients who had diabetes mellitus, high myopia, and uveitis were excluded.

### Image acquisition protocol for OCTA

A 3 mm × 3 mm OCTA macular scan was obtained using the Optovue RTVue XR Avanti with the software AngioVue OCTA (Optovue Inc., Fremont, California, United states). The software also included an artifact removal function. Images were analyzed with automated projection artifact removal. Images with weak signals (signal strength index <60) or an OCTA motion artifact score of 4 were excluded.

The image analysis of the superficial vascular plexus (SVP) and deep vascular complex (DVC) was performed with AngioAnalyticTM software. The SVP starts from the inner limiting membrane (ILM) and ends at 9 μm above the junction between the inner plexiform layer and the inner nuclear layer (IPL–INL), while the DVC comprises the section between 9 μm above the IPL–INL junction and 9 μm below the outer plexiform layer and outer nuclear layer (OPL–ONL) junction (13). Potential artifacts and segmentation errors due to the distortion of the retina were carefully inspected in each layer. The segmentation errors were manually corrected by an expert (GS) when there was intraretinal fluid in nAMD. The FAZ area, perimeter, and FD-300 were obtained with the software automatically. FD-300 shows the vessel density starting from ILM and ending at 9 μm below the OPL–ONL junction in a 300 μm wide region around FAZ.

Manual correction for the segmentation error was required in approximately half of the eyes. The correction was performed by a senior ophthalmologic resident (WHH) and confirmed by a retinal specialist (LY).

### Statistical analysis

Statistical analyses were conducted using SPSS software (IBM Corp., NY, United states; version 26.0). Continuous variables were described as mean ± standard deviation. All best corrected visual acuities (BCVAs) were converted to logarithms of the minimal angle of resolution (logMAR) before data analyses. Student’s *t*-test and one-way analysis of variance (ANOVA) were conducted for continuous variables among and between different groups after normal distribution confirmation using the Kolmogorov–Smirnov test. The Kruskal–Wallis tests were used for non-normally distributed data. Fisher’s exact tests were used for categorical variables. The Kendall tau correlation coefficient was used to examine the correlations between the OCTA parameters and different groups. A *p*-value of <0.05 was considered statistically significant.

## Results

In total, 42 eyes of 41 patients were included in this study. The mean age was 66.0 ± 7.3 years, and most patients (71.4%) were male. There were 21 eyes with exudative AMD and 21 eyes with non-exudative MNV. There were 16 eyes with type 1 MNV, 4 eyes with type 2 MNV, and 1 eye with type 3 MNV in exudative AMD eyes. In total, 20 eyes of 20 age-matched healthy subjects without retinal pathology were included as normal controls. All the subjects were Chinese and treatment naïve. Visual acuity in eyes with non-exudative MNV was superior to those with exudative AMD (logMAR BCVA 0.34 ± 0.20 vs. 0.81 ± 0.14, *p* < 0.001). There were no significant differences in age, sex, refractive error, axial length, lens status, or IOP between eyes with exudative AMD and eyes with non-exudative MNV and normal controls (Table 1).

**Table 1:** Demographic and clinical characteristics.

	Exudative AMD	Non-exudative MNV	Controls	*p*-value
Eyes, *n*	21	21	20	
Age (mean ± SD)	67.8 ± 6.3	64.2 ± 8.0	65.8 ± 6.7	0.368^a^
Sex (male)	75.0%	66.6%	70.0%	0.938^b^
BCVA (logMAR)	0.81 ± 0.14	0.34 ± 0.20	0.04 ± 0.05	<0.013^a^
Rx (diopters)	+0.30 ± 0.87	+0.14 ± 0.89	−0.07 ± 0.83	0.668^a^
AL (axial length)	23.21 ± 1.08	24.43 ± 0.99	23.42 ± 1.18	0.663^a^
Lens (phakic)	47.6%	61.9%	40.0%	0.395^b^
IOP (mmHg)	13.8 ± 2.7	14.9 ± 2.7	15.5 ± 2.8	0.587^a^

AMD, age-related macular degeneration; SD, standard deviation; BCVA, best-corrected visual acuity; Rx, refractive error; AL, axial length; IOP, intraocular pressure.

a Kruskal–Wallis test.

b Fisher’s exact test.

**Figure 1:**
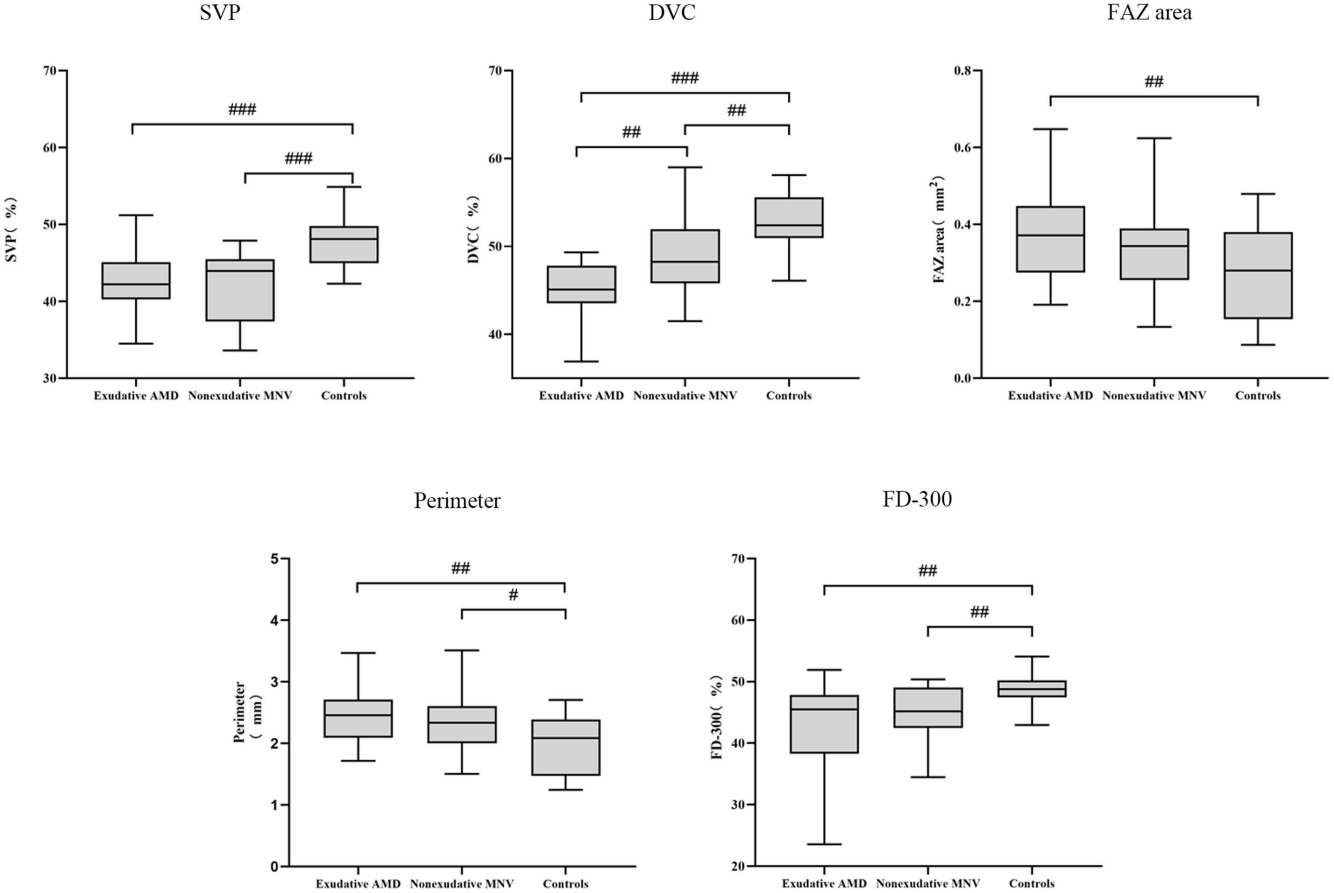
Box plot showing retinal vascular indexes in exudative AMD patients, non-exudative MNV patients, and healthy controls. #, *p* < 0.05; ##, *p* < 0.01; and ###, *p* < 0.001.

**Figure 2:**
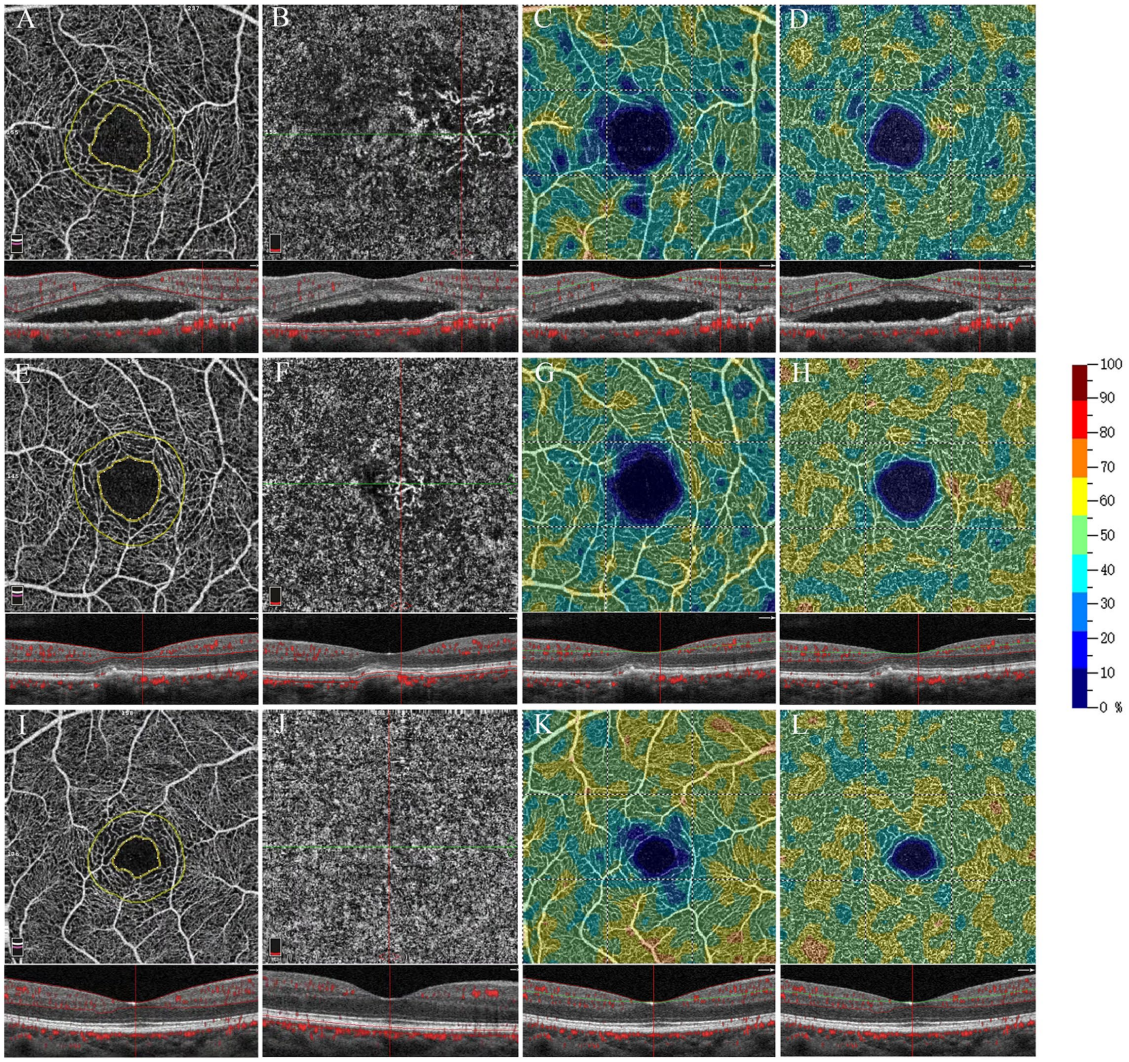
Optical coherence tomography angiography 3 × 3 mm scan of exudative AMD eyes **(A–D)**, non-exudative MNV eyes **(E–H)**, and healthy eyes **(I–L)**. The OCTA map shows the contour (inner yellow ring) of the FAZ and a 300 μm wide area (limited by the outer yellow ring) where the flow density (FD-300) was calculated **(A,E,I)**. En face OCTA and OCTA B-scan with flow marked in red **(B,F,J)**. En face OCTA angiograms with color-coded VD (color bar: warmer colors representing the higher VD) of the retinal vascular plexuses: SVP **(C,J,K)**, DVC **(D,H,L)**.

For SVP metrics, no VD differences among sectors were observed between eyes with non-exudative MNV and exudative AMD. VD of the SVP was lower in eyes with non-exudative MNV and exudative AMD compared with normal eyes (all *p* < 0.001). Conversely, VD of the DVC in eyes with non-exudative MNV was significantly greater than that in eyes with exudative AMD (*p* = 0.002). VD of the DVC in eyes with non-exudative MNV was significantly lower than that in normal eyes (*p* = 0.006) (Figures 1, 2 and Table 2).

**Table 2:** Vessel density, FAZ area, and perimeter in exudative AMD patients, non-exudative MNV patients, and healthy controls.

	Exudative AMD (*n* = 21)	Non-exudative MNV (*n* = 21)	Controls (*n* = 20)	*p*-value^a^	*p*-value^b^	*p*-value^c^	Correlation coefficient	*p*-value
SVP VD (%)	42.52 ± 4.04	42.20 ± 4.48	47.76 ± 3.58	0.812	<0.001	<0.001	0.384	<0.001
DVC VD (%)	44.88 ± 3.40	49.07 ± 4.49	52.61 ± 3.11	0.002	<0.001	0.006	0.538	<0.001
FAZ area (mm^2^)	0.37 ± 0.12	0.34 ± 0.12	0.27 ± 0.12	0.388	0.008	0.075	−0.245	0.016
Perimeter (mm)	2.47 ± 0.47	2.36 ± 0.49	1.98 ± 0.47	0.474	0.002	0.018	−0.303	0.003
FD-300 (%)	43.32 ± 6.54	44.70 ± 4.33	48.69 ± 2.45	0.452	0.001	0.001	0.340	<0.001

SVP, superficial vascular plexus; VD, vessel density; DVC, deep vascular complex; AMD, age-related macular degeneration; MNV, macular neovascularization.

a Exudative AMD versus non-exudative MNV.

b Exudative AMD versus controls.

c Non-exudative MNV versus controls.

For the FAZ area, there was no significant difference between eyes with exudative AMD and non-exudative MNV. The FAZ area was significantly larger in eyes with exudative AMD compared with normal eyes (*p* = 0.008). It was also larger in eyes with non-exudative MNV than in normal eyes, although without statistical significance (*p* = 0.075). The FAZ perimeter was smaller in normal eyes than that in non-exudative MNV and exudative AMD eyes. There was no significant difference in perimeter between eyes with non-exudative MNV and eyes with exudative AMD. Regarding FD-300, there was no significant difference between eyes with non-exudative MNV and eyes with exudative AMD. FD-300 was lower in eyes with non-exudative MNV and exudative AMD than in normal eyes (all *p* = 0.001) (Figures 1, 2 and Table 2).

In Kendall tau correlation coefficient analysis, we observed significant correlations between different groups and OCTA parameters. Retinal vessel density measured in FD-300, SVP, and DVC decreased significantly from normal eyes to eyes with non-exudative MNV and then to eyes with exudative AMD (all *p* < 0.001). The FAZ area and perimeter showed a significant increase from normal eyes to eyes with non-exudative MNV and then to eyes with exudative AMD (FAZ area: *p* = 0.016, perimeter: *p* = 0.003) (Table 2).

## Discussion

In this retrospective study, we explored retinal vessel features in patients with non-exudative MNV using OCTA. Overall, we found the reduction in retinal vessel density, especially in the DVC, might be involved in or be accompanied by non-exudative MNV activation and should be closely monitored during follow-up.

Subclinical non-exudative MNV was reported first in cadaveric eyes with AMD in the 1970s. Its risk of developing exudation in eyes with non-exudative MNV was much higher compared with eyes without these lesions, especially when the fellow eye already suffered from exudative AMD (4, 5, 11). OCTA as a non-invasive tool can be used to detect and monitor non-exudative MNV at follow-up visits (4, 14–16). Previous studies have tried to identify predictive activation biomarkers of subclinical MNV, but mainly focusing on the feature of MNV itself (5, 10–12). To figure out whether the feature of retinal vessel density of non-exudative MNV is associated with the progression to exudations, we compared retinal vessel density and FAZ parameters in non-exudative MNV, exudative AMD, and normal eyes using OCTA. To the best of our knowledge, there have not been any formerly published studies comparing retinal microvascular characteristics of non-exudative MNV with that of exudative AMD and normal control.

In this study, VDs of the SVP and DVC were lower in eyes with exudative AMD compared with normal eyes. The results are consistent with the previous studies (17–19). Not only exudative AMD eyes, significant loss of superficial vessel density and a trend for deep vessel density were also reported in intermediate AMD patients (20, 21). The alteration of retinal vessels was also noticeable in early AMD eyes where the fellow eye developed exudative AMD (22). Furthermore, it was found that AMD eyes with GA present more rapid loss of retinal vessel density and FAZ enlargement over 2 years (23). These findings support the hypothesis that retinal change can be involved in or be accompanied by the progression of AMD. Thus, it is of great importance to compare retinal microvascular characteristics between non-exudative MNV and exudative AMD.

Our study demonstrates that the VD of the DVC in eyes with non-exudative MNV was significantly greater than that in eyes with exudative AMD and significantly lower than that in normal eyes. However, there was no statistical difference in SVP VDs between exudative AMD and non-exudative MNV eyes. In correlation coefficient analysis, retinal vessel density measured in the SVP and DVC decreased significantly from normal eyes to eyes with non-exudative MNV and then to eyes with exudative AMD. Colantuono et al. found that perfusion density in the DVC decreased over time, which was lower in non-exudative MNV eyes than that in the eyes of healthy controls and intermediate AMD with non-significant differences (24). It might be explained by the small sample size that led to a relatively low statistical power. According to the study by Toto et al., superficial vessel density decreased significantly in the intermediate AMD, while no significant difference in deep vessel density was found between early AMD, intermediate AMD, and controls (20). Therefore, we suppose that the decreased flow in SVP might be earlier than DVC in the AMD course, and the further decreased flow in DVC might be related to the exudation of non-exudative MNV.

Pathologists Grossniklaus and Green first proposed that type 1 MNV might be beneficial (25), and these vessels might supply oxygen to the ischemic outer retina and protect against the progression of geographic atrophy (7, 9, 12). Therefore, we suppose that non-exudative MNV may be a protective factor for the outer retina and DVC, while for the inner retina and SVP, it may be less protective. Then, the reduction in DVC and ischemic outer retina might induce the activation of non-exudative MNV, which may impair the outer and inner retina, and lead to the thinning of retinal layers and further decreased flow in retinal vessels eventually. Thus, the reduction in retinal vessel density, especially the VD of the DVC, might be involved in or be accompanied by non-exudative MNV activation and should be closely monitored during follow-up.

It was suggested that post-receptor retinal neuronal loss could have induced retinal vascular change in AMD (23). Relative sparing of the radial peripapillary capillary plexus and impairment of underlying retinal vasculature, supporting potential anterograde transsynaptic degeneration in intermediate AMD. These findings supported the hypothesis that decreased flow in retinal vessels may be a response to a reduced metabolic demand due to thinning of the IPL and ganglion cell layers (GCLs) in non-exudative AMD (26–28). Whether the activation of non-exudative MNV follows this hypothesis remains uncertain. In this study, the reduction in retinal vessel density, especially the VD of the DVC, might be involved in or be accompanied by near-future exudation, and a prospective study of non-exudative MNV should be performed to determine whether VD of DVC is the predictive factor for the activation of MNV. The main limitation of our study is that the retinal layer thickness was not obtained. Assessment of retinal layer thickness should be implemented in the future to determine the relationship between the reduction in retinal vessel density and the change in retinal layer thickness.

We found that the FAZ area and its perimeter in exudative AMD were significantly larger than that in normal eyes. A significant increase in perimeter and a trend in FAZ area were observed in eyes with non-exudative MNV compared with normal controls. It was reported that the area and perimeter of the FAZ in eyes with non-exudative AMD were larger than those in the normal controls (29). Qiu et al. reported that the FAZ area did not change much in exudative AMD eyes compared with the healthy controls. The possible reason for the disagreement is that OCTA was performed in different scan patterns. FD-300, as an OCTA biomarker, comprised segmentation of SVP and DVC, which in diabetic macular edema, branch retinal vein occlusion, and retinal arterial occlusion, was lower than the control group (30–32). To the best of our knowledge, this is the first study to investigate FD-300 in exudative AMD and non-exudative MNV. In our study, FD-300 was lower in eyes with non-exudative MNV and eyes with exudative AMD than that in normal eyes, and there was no significant difference between eyes with non-exudative MNV and eyes with exudative AMD. In correlation coefficient analysis, FD-300 decreased significantly from normal eyes to eyes with non-exudative MNV and then to eyes with exudative AMD. This evidence also supports that the reduction in retinal vessel density might be involved with or be accompanied by the progression of AMD and the activation of non-exudative MNV.

### What is the current set of recommendations in protocols?

The diagnosis of symptomatic exudative MNV in AMD should be made on the basis of medical history and the use of multimodal imaging technologies, potentially including OCT, FA, and ICGA. Current expert consensus statements support the use of anti-angiogenic therapy as the first-line treatment of patients with nAMD. The benefits of intravitreal injections are not only to halt the progression of the neovascular component of the disease but also to improve visual acuity and quality of life.

Based on multimodal imaging technologies including OCT, FA, and ICGA, symptomatic exudative MNV in AMD was detected. The use of anti-angiogenic therapy as the first-line treatment of patients with nAMD has been fully supported by expert consensus statements (33–35). The utility of intravitreal injections is not only to halt the progression of the disease but also to increase visual acuity and quality of life as much as possible. Although no controlled clinical trial has been performed to provide definitive recommendations, for subclinical MNV which are asymptomatic in treatment-naïve eyes, the management of such lesions is not yet codified, and most of the authors agree not to treat them in the absence of fluid on OCT (6). They should be closely monitored to detect early signs of exudation that may affect visual function (4). To avoid delayed treatment or overtreatment, it is of great importance to find out the characteristics of non-exudative MNV that could serve as predictors of near-future exudation. Previous studies mainly focused on the features of MNV itself (5, 10–12). In this study, we found that the reduction in retinal vessel density, especially the VD of the DVC, might be involved in or be accompanied by near-future exudation and should be closely monitored during follow-up in order to ensure prompt anti-angiogenic therapy. Additionally, this study falls within recent recommended approaches to healthcare research (33–39).

## Recommendations for evidence-based practice

One key insight into this study is that instead of prophylactic treatment, non-exudative MNV should be closely monitored to detect early signs of exudation. In this study, we found that the reduction in retinal vessel density, especially the VD of the DVC, might be involved in or be accompanied by near-future exudation. Future studies are necessary to test the hypothesis in a prospective manner. Assessment of retinal layer thickness should be implemented in the future to determine the relationship between the reduction in retinal vessel density and the change in retinal layer thickness.

In conclusion, this exploratory study investigated retinal microvascular characteristics of non-exudative MNV, wherein the presence of this condition was associated with a reduction in retinal vessel density, especially in the DVC, and that further reduction in the vascular density of the DVC may be associated with neovascular exudation. Consequently, monitoring of DVC vessel density may help with risk stratification and determination of appropriate follow-up intervals, thereby ensuring prompt access to anti-angiogenic therapy.

## Limitations

Our study has several limitations. First, despite the retrospective study, the study is exploratory, and the resources to undertake a clinical trial were not available. In addition, further studies are necessary to test the hypothesis in a prospective manner. The second limitation is that the retinal layer thickness was not obtained. Assessment of retinal layer thickness should be implemented in the future to determine the relationship between the reduction in retinal vessel density and the change in retinal layer thickness. The third one is that approaches to enhance the quality of OCTA images and reduce noise were not used because it is a retrospective study. Future studies should reduce noise in OCTA images. Another limitation was that the effect of intraretinal fluid on measurements of VD is not well understood.

## Data availability statement

The original contributions presented in the study are included in the article/supplementary material, further inquiries can be directed to the corresponding author.

## Author contributions

YG and SZ conceptualized and wrote the article. YZ, TY, and GS undertook the activities associated with the review and meta-analysis. PM revised the whole article and methodology. All authors contributed to the article and approved the submitted version.

## Figures and Tables

**Table:** 

MNV	macular neovascularization
AMD	age-related macular degeneration
OCTA	optical coherence tomography angiography
VD	vessel density
SVP	superficial vascular plexus
DVC	deep vascular complex
FAZ	foveal avascular zone
CNV	choroidal neovascularization
FA	fluorescein angiography
ICGA	indocyanine green angiography
ILM	inner limiting membrane
IPL	inner plexiform layer
INL	inner nuclear layer
OPL	outer plexiform layer
ONL	outer nuclear layer
BCVAs	best corrected visual acuities
logMAR	logarithm of the minimal angle of resolution
ANOVA	one-way analysis of variance
